# Infertility as Trauma: Understanding the Lived Experience of Involuntary Childlessness

**DOI:** 10.1007/s11013-024-09871-7

**Published:** 2024-07-14

**Authors:** Cristina Archetti

**Affiliations:** Department of Media and Communication, University of Oslo, P.O. Box 1093, Blindern, 0317 Oslo, Norway

**Keywords:** Infertility, Trauma, Involuntary childlessness, Experience, Moral agency, Psychotherapy, Identity-Oriented Psychotrauma Theory (IoPT)

## Abstract

Infertility, to those who are affected by it, is much more than whether one manages (or not) to have a child: it can be a traumatizing experience. Based on a clinical case study that involved one-to-one psychotherapy sessions and semi-structured interviews with six involuntarily childless women living in Norway, this article develops the argument that there is a need to treat infertility as trauma, both conceptually and from the perspective of therapeutic practice. The analysis contributes to our understanding of trauma as a disruptive event that erodes a person’s moral agency. It does so by outlining conceptual and therapeutic tools that illuminate what happens in the psyche as a result of the trauma: they help explaining why the moral agency of different individuals is damaged to different extents, and how therapy can repair it. In relation to the issue of involuntary childlessness, the analysis shows where infertility fits within one’s traumabiography—a map of the way adverse experiences over the life-course have affected one’s psyche and behavior—both as traumatizing in itself and connected to previous traumas. This understanding enables more effective therapeutic support and better care for many individuals whose long-term suffering would otherwise remain unacknowledged and untreated.

## Introduction

Involuntary childlessness, i.e., not having children not by choice, is a growing, yet little understood phenomenon when one moves beyond its purely demographic and medical dimensions. The argument developed in this article is that there are painful relational and existential dimensions to unfulfilled parenthood and that the current inability to appreciate them, both in research and in therapeutic practice, is an obstacle to providing better care and support to those who need them. On average, one woman (over 45) in five (Beaujouan et al., 2017, p. 4; OECD, 2015, p. 5) and one man in four in the Western world (Präg et al., 2017, p. 8) have no children. In countries like Australia, Italy, Germany, or Japan, practically every fourth woman born in the 70s will end her reproductive cycle without children (Beaujouan et al., 2017, p. 4; Ford et al., 2002, p. 29; OECD, 2015, p. 5; The Economist, 2017). It is estimated that 90% or more of all individuals without children are childless as a result of infertility rather than a deliberate choice (Keizer in NWO, 2010; Tanturri et al., 2015, p. 33).^1^

Despite the extent to which involuntary childlessness affects our societies (Kassam et al., 2015; Shaw, 2023), its reality is shrouded in silence (Archetti, 2020, 2021b). In fact, even in democratic countries that enjoy freedom of expression, infertility is a taboo (Pfeffer & Woollett, 1983, p. 82; Thorn, 2009, p. 48). The well-documented stigma associated with it (Riessman, 2000; Whiteford & Gonzalez, 1995; Yeshua-Katz, 2019) also affects research, to the point there is a chronic neglect for the anthropology of infertility (Inhorn, 1994), particularly the lived experience of involuntary childlessness (for rare exceptions see: Archetti, 2020, 2021a; Fieldsend, 2019). This study is a contribution to the anthropology of infertility and a response to calls for more qualitative experience-based studies of it (Schwerdtfeger & Shreffler, 2009, p. 215).

“Infertility” is here approached beyond the medical definition provided by the World Health Organization (WHO, 2022) as ‘a failure to conceive after regular unprotected sexual intercourse for one year.’ While childlessness might be related to medical problems—rarely to outright sterility (Beaujouan et al., 2017, p. 4)—it is in most cases a manifestation of “social infertility” (Berrington, 2016, p. 58) or “childlessness by circumstance”: life turned out this way due to a myriad reasons, like having been single, having suffered the death of one’s partner, having been ill during fertile years, or not being able to afford assisted reproduction to name the most common (Day, 2013). The investigation of such more broadly conceived range of infertility experiences is meant to reclaim involuntary childlessness as a subject of study in its own right, beyond its current narrow understanding as a *reproductive*—and, in that respect, *bodily*—disfunction, missed parenthood, or “non-event.” Infertility, instead, is conceived here as a complex experience with far-reaching repercussions on all domains of one’s life—as we will see, from identity to relationships, health, sense of belonging (or lack of it) in our communities and society at large, and existential issues (Archetti, 2020).

A number of studies have established a link between infertility and trauma (Bartlik et al., 1997; Bradow, 2012, for a couple of examples). There is, in fact, strong evidence that infertility can have devastating consequences on the affected individuals’ mental and physical health in the long term (for instance: Tsigdinos, 2022; Wirtberg et al., 2007). Yet, these insights do not appear to make any practical difference to the care of those affected, whose suffering remains, largely, not only untreated and unacknowledged (Archetti, 2019), but also even actively dismissed (Day, 2021). My aim is to make a case for taking infertility as trauma seriously.

I draw on the breadth of perspective I matured as long-term researcher of the experience of involuntary childlessness, psychotherapist trained in Identity-Oriented Psychotrauma Theory (IoPT), and infertility survivor. The issues addressed by the analysis especially arise from having studied the existential aspects of the infertility experience of the permanently childless, i.e., those who remain without children throughout their life. It particularly struck me that so many of those I have been talking to over the years and who had been unable to conceive wished (in their words, now paraphrased) to go back to the life they had “before infertility.” They wanted to “move on.” Yet, they remained stuck in grief, were exhausted by having to navigate constant triggers, often many years after the end of their fertility journey, did no longer “feel like themselves.” The questions I wanted to find an answer to and which stimulated this investigation were as follows: Why is it so difficult for some to let go of the plan of having a child? Why is it so problematic, and for some not possible at all, to fully return to a “normal” life? Through my research and first-hand experience, I have also become deeply aware of the lack of support structures for those who are worst affected in the long term. The further issue I wanted to address was thus: How can we improve our understanding of the suffering caused by involuntary childlessness and how can we provide better care for it?

My answer to these questions is that to fully understand the depth of the consequences of infertility and to offer appropriate solutions we need to grasp not only what happens in one’s psyche, but also what changes in the practice of everyday life: one’s perceptions, thinking, and behavior. The argument I develop is that infertility can be, in itself, a trauma and that it can nest within previously existing traumas, often, as the analysis will reveal, from early childhood. As trauma, infertility limits the ‘moral agency’ of those affected, i.e., the ability to envisage for oneself and actually live a “good life” (Blacksher, 2002; Myers, 2016, 2019; Myers & Ziv, 2016). However, through therapy, it can be restored. In this clinical case, I outline theoretical and therapeutic tools to understand what happens in the psyche as a result of the disruptive traumatic life-event that infertility is, providing an insight into why the moral agency of some individuals is affected to different extent than others,’ as well as into how and why it can be repaired through therapy.

## Trauma and Infertility

Understanding why infertility can be a traumatic experience involves more than acknowledging that “everyone’s perspective is different.” Are there any common patterns that underpin the infertility experience? Why does trauma occur? Addressing these questions further demands two conceptual an analytical moves, which I am now going to illustrate.

First, we need to adopt a broader conceptualization of trauma. According to the definition by the American *Diagnostic and Statistical Manual of Mental Disorders* (DSM-5), trauma requires ‘actual or threatened death, serious injury, or sexual violence’ (APA, 2013, p. 271; see also Pai et al., 2017).^2^ However, trauma can and does result also from other occurrences that one might (wrongly) regard as “less extreme,” such as illness, loss, neglect, or ‘any other disturbing event’ (Roberts Stoler, 2019). When it comes to infertility, Bradow explains that, by interfering with forming a family and carrying on one’s genetic code, basic drives we have as human beings, the condition constitutes a threat to ‘expectations of life’ (Bradow in Rettner, 2012, p. n.p.; see also Bradow, 2012). Trauma, more specifically psychotrauma, is understood here as anything that overwhelms us emotionally and/or physically and that we are unable to process (Broughton, 2021; Maté & Maté, 2022; Menakem, 2017; Ruppert, 2012, 2016). Trauma, in addition to this, does not just happen in the mind. It also adversely affects our identity (who we are), our behavior (what we do), our perceptions of the world (what we think), and our body (our health) (Broughton, 2021; Levine, 2010; Maté, 2019; Maté & Maté, 2022; Ruppert, 2012, 2016; Ruppert & Banzhaf, 2018; Van der Kolk, 2015). This leads us to a second point.

Thoroughly understanding the multifaceted impact of trauma requires comprehending both conceptually and therapeutically what happens in the psyche, but also appreciating how the ‘moral agency’ (Blacksher, 2002; Myers, 2019; Swygert, 2020) of those who suffer from infertility is impacted. Moral agency is the ability to be perceived as ‘good enough’ by others so as to make possible intimate connections (Brown et al., 2023; see also Myers, 2015). This can involve, among its basic dimensions, the ability to write one’s own life story, being recognized and respected as the person one imagines to be, the capacity to aspire to and enact a “good life” (Blacksher, 2002; Myers, 2016, 2019; Myers & Ziv, 2016). Trauma, as we will see, effectively damages all of these abilities (Swygert, 2020). Although the concepts of “trauma” and “moral agency” arise from different traditions of enquiry—respectively, psychology and anthropology—their combination is analytically productive, particularly where trauma is not assumed to be confined to the mere mental domain, as in this study. “Moral agency” in my analysis, in fact, assists in tracking empirically both the damage that trauma inflicts and the benefits of its healing through therapy: while we cannot directly “see” what happens in the psyche, we can map the transformations the psychic structures undergo as they are reflected in observable changes in the moral agency of those affected.

In this study, trauma is not a discrete event that merely happens to a person at a certain point in life. It is also not necessarily a life-threatening occurrence. Instead, trauma is taken to be a continuous meaning-making process that reverberates through time (both backwards and forward), emerging in one’s constant encounter with others and the world (Lester, 2013, p. 754). Trauma, in other words, is *relational*. And it is precisely in the realm of relations that the essence of trauma resides. It is telling that the Greek root of the term trauma means “wound.” This “cut” is, effectively, the severance of the connections we have with the world we know, are familiar with, and feel safe inhabiting (Lester, 2013, p. 754). In this sense, trauma is a ‘moral breakdown’: a moment when our *morality*, our unreflective way of being-in-the-world, is challenged, we stop dwelling in the ‘comfort of the familiar’ and stand, instead, ‘uncomfortably and uncannily’ (Zigon, 2007, p. 138).

Infertility constitutes for most a devastating rapture in one’s life plans:^3^the dream of becoming a parent, often unquestioningly cultivated since one was a child, within societies that are inherently pronatalist (Brown & Ferree, 2005), is suddenly not unfolding naturally, or not materializing at all. Because very little awareness exists of the devastating repercussions of not being able to conceive, even among practitioners and therapists (Archetti, 2020, pp. 126–127, 233–234), it is worth briefly outlining their range and depth. They all consist, effectively, in disconnections, i.e., moral breakdowns: from one’ self and life-narrative, from one’s communities of support, and from society at large. Infertility deeply affects identity (Archetti, 2020; Greil, 1991; Leon, 2010): if to be “successful,” or “happy,” as we are repeated by countless messages around us, means “having a family” or “having children,” can a childless individual ever be “realized”? Can a woman (or a man) without children be a “(real) woman (man)”? Not being able to have a child additionally often leads to grief comparable to bereavement (Day, 2016, pp. 79–123; Hooper, 2018; Thorn, 2009; Volgsten et al., 2010): not only has one’s imagined child died—indeed at every arrival of the menstrual cycle—but also one’s self as a mother/father has died, together with one’s imagined future as a family, one’s grandchildren to come, the whole world of life-milestones everyone else is going to experience and one is going to miss out on. With the difference that this bereavement might continue indefinitely because there is no body to bury and no sense of closure. Childlessness also redefines relationships: although fighting adversity can indeed strengthen bonds between people, many couples do disintegrate as result of the life crisis brought about by infertility (Kjaer et al., 2014). Further to this, when being in contact with children hurts and, one by one, most of one’s friends are lost to forming “families,” most childless individuals experience, over the years, increasing isolation (Dykstra, 2006; Wenger et al., 2000). This fuels a sense of alienation and exclusion in a society that revolves around parents and their children (Archetti, 2020, pp. 214–227).

All of these severances from “normality” lead to a radical erosion of one’s ‘moral agency’ (Blacksher, 2002; Myers, 2016, 2019), understood as the ambition, ability and resources to both envisage a “good life” for oneself and pursue it. Healing, in this perspective, consists in re-establishing the links we have lost (Lester, 2013, pp. 759–760). For Myers (2016, pp. 436–439) essential aspects of this process involve social opportunities to strengthen one’s self-respect (‘social bases of self-respect’), being recognized as a good and moral person by others (‘peopled opportunities’), and having the ability to craft and edit one’s self narrative (‘autobiographical power’). Returning to involuntary childlessness, a number of studies have established a link between infertility, trauma and PTSD^4^ (Bartlik et al., 1997; Bradow, 2012; Corley-Newman, 2017; Schwerdtfeger & Shreffler, 2009; Tsigdinos, 2022). While this literature does not refer explicitly to moral agency, it shows that infertility has such severe psychological and physical repercussions on the lives of the individuals affected that, de facto, it limits their ability to envisage and practically lead a “good life” in their communities. The symptoms Bartlik et al. (1997) present by discussing three case studies of patients who developed PTSD as a result of infertility, for example, range from deriving ‘little pleasure from social contact,’ and a sense of ‘estrangement’ from other people, to difficulty in concentrating, becoming ‘obsessed’ about bodily sensations, having ‘distressing dreams,’ a sense that life is ‘on hold, indefinitely.’

To understand how long-lasting these adverse effects can be, Schwerdtfeger and Shreffler (2009) find that childless women who had experienced pregnancy loss or failure to conceive reported the ‘lowest life satisfaction and highest levels of depression’ despite a considerable period of time (7 years, in the case of their study) since the loss or missed conception (Schwerdtfeger & Shreffler, 2009, p. 211). Another investigation, which involved interviews with Swedish women, demonstrates the repercussions of failed IVF treatment as long as 20 years later (Wirtberg et al., 2007). Not only, according to the authors’ findings, were half of the women who participated in the study separated at the time the interviews took place, they also developed a low sense of self-esteem and a feeling of inferiority to other women.

All of these studies present a range of limitations. First those investigations that explicitly refer to “trauma” do so in terms of *reproductive* trauma and this, in turn, tends to be connected to a *pregnancy loss* (Bhat & Byatt, 2016, p. 1; Swift et al., 2022, p. 171). What about the adverse effects that do exist and result from never having been pregnant at all? Second, they all invariably regard infertility based on the WHO (2022) definition as a failure to conceive after regular unprotected *sexual intercourse* (Aiyenigba et al., 2019, p. 76, for one example). What of infertility, and possible related trauma, that arises from not having a partner in the first place? Estimates of the prevalence of infertility (Boivin et al., 2007) tend to rely on surveys involving women ‘in married and consensual unions’ and those seeking infertility medical care (ibid.: 1507). From the perspective of such specific (and very reductive) criteria, it is estimated that 60-80 million couples are affected by infertility worldwide (Boivin et al., 2007; Wdowiak et al. 2016 in Roozitalab et al., 2021, p. 282). This is only the tip of the iceberg. There are so many more cases than those captured by the statistics: because those affected by infertility (including men) never went through the medical system or attempted IVF (as one of the participants in my study), or because they constitute cases of “social infertility” (the latter might also include both singles and same-sex couples). The final limitation is that we do not know why infertility affects different individuals to different extents. Considering how many million people are affected worldwide and how destructive the consequences of infertility can be on the daily ability to function of those affected, potentially for many years, it could not be more urgent to treat infertility with the attention, seriousness, but above all, the *care*, that it deserves.

## Infertility and Psychotrauma Theory

After having illustrated the broader understanding of trauma that informs this study, I am zooming in on how psychotrauma is approached within the empirical clinical study. IoPT and the work of its founder, Franz Ruppert, have a prominent role in this theory section and, later, in the analysis. Not only is IoPT the approach I trained in as psychotherapist and the interpretive framework I applied in the psychotherapy sessions. IoPT theory also helps explaining what happens in the psyche that leads, first, to a ‘moral breakdown’ (Zigon, 2007)—i.e., what trauma translates into in the practice of everyday life—and, later, to moral agency repair through therapy.

A psychotrauma arises from a situation one is unable to deal with through the mental and physical capacities one has at any given time (Menakem, 2017; Ruppert & Banzhaf, 2018, p. 24). It is an overwhelming experience we might undergo as a result of something that *happens* to us—an accident, a loss—but also something that did *not happen*—a parent who did not take care of our basic needs or the dream of having a child remaining unfulfilled, for instance. If we are not able to respond to the overwhelming circumstances by removing ourselves from the situation—*flight*—or directly addressing the cause of the threat—*fight*—in order to survive we either *freeze* or submit (*fawn*) (Torsheim, 2022). A part of our psyche, in practice, splits off and gets “buried” into our unconscious, where it will continue to live without us either realizing it is there and/or being able to reach it.

An experience is traumatizing when we feel we have no control and feel helpless, when our identity, will, our needs or our sense of integrity are threatened (Lester, 2013, p. 756). This can happen as a result of a sudden, unexpected and catastrophic event, like a natural disaster or a physical aggression, but can also unfold over time (Broughton, 2021, pp. 71–73). Undergoing fertility treatments that are invasive, humiliating and dehumanizing (Tsigdinos, 2022) and where one has no control, neither of the procedure nor of the result, means one is stuck in a situation of extreme uncertainty, physical and mental exhaustion, and, when the efforts lead to no result, desperation, often for years on end.

**Fig. 1:**
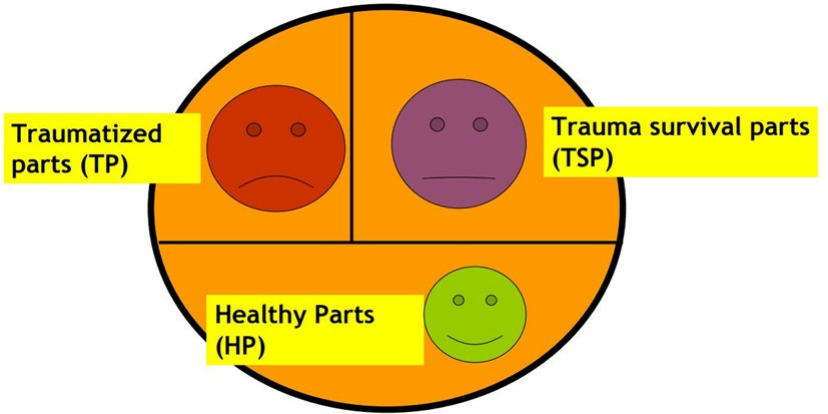
Splitting of the psyche after traumatization (Ruppert, 2012, p. 69)

For Ruppert, as a result of trauma, our psyche presents three kinds of parts: ‘traumatized,’ ‘survival’ and ‘healthy’ parts (Fig. 1). The ‘traumatized parts’ are frozen in the original trauma situation: they are locked in the unconscious and there they continue to live, feel, and think as when the trauma first occurred (Ruppert & Banzhaf, 2018, p. 26). They also always present the same age and developmental stage as the person had at that time. These are the parts that might get triggered by reminders (situations, sounds, words, smells, bodily sensations) of the original trauma (Ruppert, 2012, pp. 72–73). The ‘survival parts’ serve the function of protecting us from any memory of the original trauma through, for instance, telling themselves that ‘it wasn’t so bad’ (denial of trauma) or distracting themselves with all sorts of “strategies,” such as burying themselves in work (Ruppert, 2012, pp. 73–83; Ruppert & Banzhaf, 2018, p. 27). The ‘healthy parts’ are those that are able to respond appropriately to reality, to process it and to understand it in its complexity (Ruppert & Banzhaf, 2018, p. 25). While the traumatized parts experience the world from an emergency- and crisis perspective, the healthy parts engage with the world with curiosity and confidence in their own resources in meeting changing situations and potential challenges (Ruppert, 2012, pp. 70–72).

Although trauma can happen at any time in our life, it is far more likely to become overwhelmed when we are extremely young and completely dependent on the care (or lack of it) of our parents: from the time of conception, through our birth and in early childhood (Ruppert, 2016). In this respect, Ruppert envisages a hierarchy of four kind of traumas, where early existential traumas become the ground for the possibility of later ones: identity-, love-, sexuality- and self-inflicted trauma. ‘Identity trauma’ refers to the trauma experienced, still inside the womb, by a child who is not wanted by the mother (Ruppert, 2020, pp. 100–108). This is not necessarily the result of a deliberate rejection by the mother. Rather, it can be unconscious. Perhaps the mother is not physically or psychologically ready for a pregnancy or the mother is traumatized and therefore unable to be fully present (again physically and mentally) to welcome her child into the world. ‘Love trauma’ is what a child experiences when s/he is not loved in the way s/he needed, perhaps by not receiving physical warmth or expressions of affection by the parents (Ruppert, 2020, pp. 109–114). ‘Sexuality trauma’ relates to any form, direct or indirect, of sexual abuse (Ruppert, 2020, pp. 115–151), but also, more broadly, to a violation of boundaries. As Kersten explains (2018, p. 305) there is a wide spectrum of situations that might qualify as such, but what makes the difference is how the person *experiences* the violation. In this perspective being forced to eat, for example, or being pushed into roles that are not appropriate for one’s age, as in the case of a small child caring for one’s own adult parents, could be a physical and psychological violation of boundaries. ‘Self-inflicted trauma’ happens when a person who has been a victim of trauma, as a survival strategy, in order not to feel the pain and unbearable emotions of the traumatized parts, turns into a perpetrator to protect her/himself from the possible shame, humiliation, terror, anger the original aggression caused (Ruppert, 2019, Chapter 5: 91–131). It can involve inflicting trauma on others—this is traumatizing because there is always a healthy part inside us that knows that this is wrong: we feel shame and guilt—but also inflicting trauma on oneself.

According to Ruppert (2021, pp. 14–15) a healthy psyche can distinguish between ‘I, you and we,’ ‘past, present and future,’ ‘realities and illusions,’ ‘the possible and the unattainable.’ In other words, and returning to the issue of involuntary childlessness, if having a child is not possible or the sacrifices for one’s own mental or physical health, or impact on one’s relationships become disproportionate in relation to the potential outcome, a healthy individual should be able to act and make choices accordingly. In the therapy context presented here, addressing/healing the trauma of infertility practically means coming to terms with a life without children. More specifically, that having a child is either not possible, or that more attempts come at a cost—and is one willing, freely and having considered all options, to pay that price?

## Method and Psychotherapy Practice

The clinical study I present is phenomenological (Desjarlais & Throop, 2011) in two respects. First because it places at the center of the analysis the experience of those who are themselves affected by infertility. Second because it is attuned both to the details of individual circumstances—the features of personal stories that will later feature in the ‘traumabiographies’—and the broader, general trends that transcend each person’s specific situation—why and in which circumstances some are more affected than others by infertility.

It involved six involuntarily childless women aged 36 to 49. Five of them are Norwegian, one is originally from a Southern European country. Three live in the Oslo region, and three reside in the North of the country. Two were recruited through a closed Facebook page for permanently childless women the author established in 2019 (65 members); three through an ad published on the monthly newsletter of the organization *Ønskebarn*, a Norwegian interest organization for the involuntarily childless (about 400 members); one joined via the contact network of a therapist colleague.^5^

Five of the participants underwent fertility treatment (IVF) for between three and 6 years (four to nine cycles). Three of them ended IVF between 6 months to 1 year before the time of the interview, one stopped 3 years earlier. One participant had ended her last treatment three weeks before the interview and later decided to continue with more attempts. One of the women never went through IVF and had been waiting for adoption for 4 years at the time of our first conversation. I ran 2 to 3 one-to-one IoPT therapy sessions (Ruppert, 2019, pp. 165–167) with each of them (16 in total) at intervals of 6–8 weeks. I also conducted semi-structured individuals interviews before and after the cycle of psychotherapy sessions (12 in total). All therapy sessions and interviews took place between June and November 2022.

An IoPT therapy session revolves around an “intention”: a statement, a question, or even a drawing of what the person undergoing therapy (“intention giver”) wishes to investigate, explore, and find clarity about. The therapist is a facilitator in this process, providing the framework where the exploration can take place safely and systematically. In the one-to-one session, this happens by asking the intention giver to select a maximum of three elements from the initial statement/question. The intention giver then “resonates,” in turn, the elements in the order she prefers. “Resonance” is the process through which the intention giver, through the images, thoughts, physical sensations that each element elicits, taps into the implicit memory of the body (Gendlin, 2003; Levine, 2010), thereby coming into contact with parts of oneself that are not normally consciously accessible. These might be traumatized parts, survival parts, or healthy parts. The resonance provides the opportunity to explore, through the guidance provided by the therapist, when the traumatized and survival parts formed, what happened at that point in the life of the intention giver, particularly which needs were not met.

**Fig. 2:**
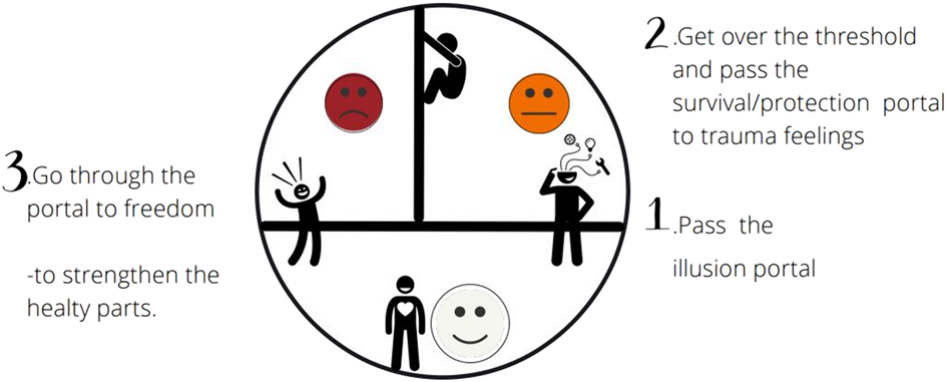
Integration of the splits in the psyche after a trauma experience (Ruppert, 2014, p. 275)

Acknowledging the reality (‘I do not feel good enough [because I am not a mother],’ for instance) and the unmet needs (‘I need understanding [of what I am going through]’) is a first step in breaking through the ‘illusion portal’ created by the survival parts (‘I am fine’). This further leads to passing the ‘threshold’ of ‘protection’ of the trauma feelings. By engaging with, i.e., *feeling,* the emotions and pain the intention giver found too overwhelming in the past, the therapy session starts a process of reintegration of the traumatized part, ultimately allowing the intention giver to move toward ‘freedom’ (i.e., expressing one’s authentic identity and will—restoring one’s ‘moral agency,’ in other words) and strengthening one’s healthy parts (Fig. 2).

In this study, the therapy sessions enable to chart the ‘roots of the trauma biography’ (Ruppert, 2019, p. 168). The ‘traumabiography’ is a map of the way adverse experiences have affected over the life-course one’s psyche and behavior (Ruppert, 2020, pp. 168–170). As such, they have both a therapy and a research function. As therapy, they allow to reintegrate parts of the intention giver that have split off. In terms of research, they enable the therapist-researcher to identify the traumas that, within the unique life history of each of the women, are potentially behind their infertility experience and whether infertility is, in itself, a trauma—outlining a ‘traumabiography’ (Ruppert, 2020, pp. 168–170).

The aim of the interviews was twofold. First to get a sense of the women’s personal journeys through life and where they stood in relation to the experience of infertility before the therapy. Questions during initial interviews included why they decided to take part in the study, what was their “story,” what they currently struggled with in their everyday life: effectively what it meant and how it felt, concretely, for each of them, to be childless; what they were hoping to achieve through the therapy. Second, the final interviews wanted to find evidence that might suggest that: (a) a reintegration of traumatized and survival parts was taking place or had at least initiated, and (b) the participant had achieved her objectives and/or benefited in other way (if so, how) by the therapy.

The point of IoPT therapy is the reintegration of the split off parts, restoring the integrity of the person, her/his will, joy for life, autonomy, liberating her/him from the constraints of possible identifications with others’ traumas and motives (parents’ or society’s, for instance) (Ruppert, 2021, p. 15). This is, effectively, what the repair of moral agency consists in psychotherapy terms. Trauma, in addition to this, does not only affect the mind, but is embodied (Levine, 2010; Menakem, 2017; Ruppert & Banzhaf, 2018; van der Kolk, 2015). Survival strategies aimed at keeping uncomfortable emotions out of reach require a huge amount of energy: we might feel drained, demotivated. We also become unable to remember things, to think straight, imaginatively and to find solutions. The final interviews thus focused on concrete examples of any changes in the women’s thinking, behavior, sensations in their bodies, that they perceived as improving their autonomy, energy, creativity—their ability, in other words, to pursue and lead a “good life.” All of these, effectively, were indicators that the participants' moral agency was getting restored.

## Findings: Infertility as a Trauma Nested Within Previous Traumas

An important observation is that most traumatized and survival parts that emerged during the therapy sessions had roughly the same age of the intention givers. This means that the split off parts were fully adult, either from the present or the time the participants underwent fertility treatment. This is a first confirmation that infertility, for the participants, is a traumatizing experience in itself.

All of the women experienced (and still experience) suffering because of the many losses they have been through. They are made worse by the fear of being triggered at any time and by the sense of isolation at being surrounded by relatives and acquaintances who do not really understand their experience. Importantly, the ‘losses’ involuntarily childless individuals go through are not just the actual loss of a child, through miscarriage for instance, but the loss of a child that exists, regardless of her/his physicality, in our psyche and a whole imagined future as a parent. Three of the participants in my study, for example, were never pregnant, another had positive pregnancy tests for two weeks. I was also never pregnant. And yet (in fact, *because* of that) we all bear the consequences of trauma. In this sense, current research that refers to ‘trauma’ only in case of a ‘perinatal loss’ (Bhat & Byatt, 2016, p. 1, for one example) is based on a reductive understanding that needs to be revised and expanded.

Infertility, beyond being a trauma in itself, is also a powerful trigger on existing traumas and, along the hierarchy outlined by Ruppert—where later traumas “nest” within earlier ones—it is possible to see how deeply the roots of the infertility trauma can reach into the past, often into one’s early childhood. I will first present some examples of the kind of traumas I identified in the women’s traumabiographies. Their cumulation over time help explaining why it is so difficult to come to terms with a life without children. They also show vividly not only the mechanisms at work in the loss of moral agency, but also what this empirically consists of in the context of the participants’ lives across their life-course.

### Identity Trauma

Indicators that suggest identity trauma in the participants’ accounts are identifications with specific roles, specifically that of a mother (‘who am I if I cannot be a mother?’), or the difficulty in recognizing the right to one’s own space: most of the participants, in this perspective, struggled establishing boundaries or even recognizing they had needs at all. As Ruppert explains, an individual affected by identity trauma would perceive just wanting to be oneself as ‘impossible and self-centred’ (Ruppert, 2020, p. 105). Identity trauma also becomes the ground for love trauma and self-inflicted trauma: since all one does is ‘for others or for a higher purpose’ (Ruppert, 2020, p. 105), not for oneself, this impairs our self-love and our ability to take care of ourselves (Ruppert, 2020, p. 105). I will return shortly to further examples from the participants that illustrate these points.

### Trauma of Love

Most women in the study, as children, longed to be loved differently than their parents did. For example, in her first therapy session, Anna states that she was never loved by her father (‘I am not sure he even knows what love is’) and on two occasions tells of how he cried when she was born at discovering that she was not a boy. In her second therapy session, although she underlined that her mother did everything she could in a situation of poverty (‘she gave 200%’), she also acknowledged ‘I would have probably been happier if I had had more love from her.’ In her third therapy session, she wanted to understand why she feels intense, physical pain, which often lingers for many days, in hearing stories of child abuse or children suffering. In working with the intention “How a child’s pain can hurt me so much? [sic]” it emerged that the pain she feels is, in reality, the pain she herself experienced as a child for not being cared for by her mother in the way she would have liked to.

Marianne is angry at and hurt by comments by family members and friends who do not understand that she wants to “stop trying.” While working on the intention “I feel angry when I am sad” (third therapy session) she realizes that her emotions are related to a fear that she will not be liked and even abandoned by those she loves. That is also why she does not establish boundaries: ‘I don’t want my surroundings to be uncomfortable […] I am afraid that they are going to leave me.’ She began doing this, applying this survival strategy, when she was ‘4 or 5,’ ‘before I lost my father,’ a time when she was afraid of losing the love of her mother: ‘I liked to be as *invisible *as possible.’ In particular, she was concerned that her mother, who had divorced her father (she ‘vocally expressed how much she hated that person’) ‘would only see the father in me.’

### Trauma of Sexuality (Lack of Protection)

While I did not observe any trauma of an explicitly sexual nature, I identified violations of the physical and psychological boundaries in the traumabiographies of several of the participants. More specifically, material circumstances and the lack of protection by the parents, often because the latter struggled with their own traumas, meant that some of the women were forced into situations, roles and responsibilities that were too heavy to bear for them as small children. Marianne, for example, whom we already mentioned in relation to a trauma of love, recounts sitting in the first row at the funeral of her father when she was 6, surrounded by relatives, but not her own mother. She felt completely alone in her grief. Trying to regain some form of ‘control’ Marianne stopped eating and began suffering from anorexia at the age of 9. For Ruppert (2016, p. 253) anorexia, denying oneself food is, in terms of a love trauma, ‘an attempt to disappear physically’ by a child who is not wanted (or that is the child’s perception).

Liv was bullied when she was in 3rd grade and those events, even if they might appear remote and totally unrelated to infertility, still weigh heavily on her present. She started her first therapy session with the intention “Exploring my self-worth within myself. Realize my purpose of living [sic].” She is concerned that, if having a child does not work out, she will not know what to do (‘what the *hell* do I do then?’). While resonating “self-worth” it emerged that these feelings are, deep down, related to her school experience. At that time her grandmother, who lived with her family and whom Liv saw as a ‘third parent,’ further contributed to her insecurity. Although Liv expected her grandmother to support her ‘unconditionally,’ she felt that the woman broke her trust. On the ground that ‘they had done nothing to her,’ she invited the classmates who bullied Liv around her place. Not only did Liv feel unsafe in her own house (‘the bully was in the home’). She also did not feel like she could live up to the standards of other people, whom her grandmother tended to praise (‘what did others have that made them so good?’). This has not only affected her sense of self-worth, but has also meant she constantly has tried to adapt herself to others. She recalls how she would try out different behaviors to see if that would appease the bullies. As she describes her attitude later in life (last interview): ‘I have always been giving and giving and giving and giving and never receiving anything...I have been like a dog ...that runs around and wants everyone to love you.’ This now applies to unconsciously trying to satisfy social expectations about becoming a mother.

### Self-Inflicted Trauma

Several of the women I talked to, not differently from what I or others I have encountered have previously experienced in undergoing fertility treatment, were not in control of the process and had lost sight of why they were doing it: the IVF attempts had somehow become an absorbing goal in themselves, even leading to a sense of ‘dread’ in at least two of the women upon merely imagining the treatments would actually lead to a pregnancy. A third participant felt she was not able to ‘draw a line under it,’ even if the attempts had ended years earlier. Another stated that the “end line” would be ‘when the money finished’ rather than her deliberate choice. Relentlessly pursuing fertility treatment despite the enormous impact and objective damage on one’s life (‘I felt I was dying,’ ‘it was terrible’ repeated over and over again) is, in this perspective, not only traumatizing, but a way to inflict trauma on oneself: punishing oneself for not being ‘good enough,’ for not achieving what one ‘should,’ often for the sake of others (one’s own parents or one’s husband, for example), not for oneself. While having a child is a life-milestone and a rite of passage in virtually every society (Boivin et al., 2007, p. 1506), being *unable* to take care of oneself because one is dedicating all one’s efforts to pleasing others and conforming to social expectations is not, in an IoPT perspective, the behavior of a healthy individual. Rather, these are all possible manifestations of a trauma of identity. This also shows how the loss of moral agency brought about by infertility is *connected to* and a *further layer of* an erosion that had already occurred in the women’s lives.

Further to this point, Marianne (who, we have seen, used to suffer from anorexia) referred to repeatedly having ‘crossed so many boundaries’ with her own body: ‘I feel like I have lost myself so many times trying to please others’ (third therapy session). She has done so for her husband—’I went through more than I would have done because he really wanted it [a child]’ (first therapy session)—something she now resents him for. Adapting oneself to the needs of others is, effectively, the same survival strategy she first started pursuing to deal, as a child, with her mother.

## Discussion: What It Takes to Heal the Trauma of Infertility

Although there are individuals who do not *want* to have children, longing for a child tends to be perceived as “normal” by most. However, that natural longing might become entangled with and even be fueled by an unfulfilled thirst for love that one had as a small child. Having a baby, effectively, can become a survival strategy. What I could observe in the therapy sessions, more specifically, was that some of the women would have liked to have a child to give her/him the love they did not themselves receive.

Anna, for instance, is concerned about whether her father will like the child she might acquire through adoption. In exploring the intention “How my father will/would react when I appear with my adopted child or realise [sic] I won’t have any child?” it becomes clear that the child is a projection of herself and an opportunity for her father (who, as already mentioned, cried when she was born because she was not a boy) finally loving her. As she reflects after the end of the session: ‘Maybe unconsciously I want [him] to love the child...as a way to love me...if he loved the child, I would feel more loved.’

Nora’s words in her second therapy session further underline how having a child is a survival strategy and, ultimately, even a substitute for “being” someone. Although during the sessions very little emerged of Nora’s childhood, all of this suggests a love and/or an identity trauma: ‘what you long for…when I long for a baby […] is to be important to someone, really being needed […] And that would maybe create some sense of belonging and then having a place in the world.’ This shows how powerfully the past affects both the present and our imagined future and how important it is to be able to trace the origin of the longing for a child. It explains why it feels so difficult to let go of the dream of having a baby.

While there was no expectation that the therapy, especially only 2 or 3 sessions, would lead to any dramatic change in any of the women’s lives, all women experienced healing developments. They all point, as I am going to describe them briefly, at a receding of the survival and traumatized parts and to a strengthening of the women’s healthy parts. This meant, in the terms outlined at the beginning of this article, re-establishing some of the relational connections that the trauma had severed and the repair (at least partial) of their damaged moral agency: they can re-enter their communities (‘peopled opportunities,’ ‘social bases of self-respect,’ Myers 436-238) and take decisions that serve their needs (‘autobiographical power,’ ibid. 436–437). Such restoration of moral agency, as we will see, also consists in a state of physical well-being.

As Inger shows in her final interview, for instance, she has started taking care of herself to a greater extent than before: ‘It might sound selfish, but I’ve been thinking a little more about myself […] I’ve been thinking that when I have bad days I listen more to my body.’ Berit is able to see new possibilities for the future. At the time of the final interview she is looking for a *‘*new flame*’* in her work: she has come to realize that, as a nurse specialized in the care of new-born babies while not having children herself, she has been locked for many years in a job position that made her feel ‘less than other people.’ As she phrases her ambition to change her life: ‘there are many ways to work for money […] I am not willing to put up with it any more […] I am trying to find a new platform to stand on.’ Berit, as also Nora, signed up for grief groups organized by *Ønskebarn* (organization for involuntarily childless) in the attempt to come into contact with others in the same situation. These are important steps toward accepting the perspective of a life without children. Especially for Nora, this very notion was initially petrifying.

Most participants talked about physically feeling different. Marianne starts her final interview, for example, by saying that ‘it’s good to finally say when people ask me "how are you?"… I can actually say "fine" and "good".’ Nora describes how the ‘good feelings’ she is experiencing in relation to a new puppy she has adopted feel like pleasant ‘little bubbles' in the area ‘from the stomach and up to the throat,’ in fact in the same area where she earlier had experienced ‘painful feelings.’ Inger and Anna report actively trying to “go inside themselves” to *feel*, as they did during the therapy sessions. They have, in other words, become less afraid of their own emotions. For Anna, this means having learned to feel *all* of her emotions, not just the negative ones: ‘when I feel sadness I should let it go...feel it…how it feels and try to understand why, where it comes from ...and I do the same now with happiness.’ When I ask Anna whether she still feel as much physical pain as before when she hears stories about children suffering (an issue she worked on in her third therapy session) she explains that being ‘more aware of her feelings’ gives her more ‘control’ on them and makes her able to remain present to face problems and situations. Feeling more, makes her, counterintuitively, more ‘rational,’ as she remarks.

The therapy made a difference first and foremost by providing a safe place for the women to *own* the emotions associated with infertility and to appreciate its *reality*. This could not be further from the rose-tinted and well-meaning positive framings by medical staff, family, and friends. As Marianne talks about going through the therapy in her final interview: ‘you are the first person that I have ever spoken to about the childlessness… who did not have a medical perspective or judgment or advice...and stories about who they know that [got pregnant] ...whom I just can speak to about it *as it is.*’

Sharing one’s story in a safe setting is healing because acknowledging one’s reality and feelings as valid is the initial step for crossing the first wall built by our survival parts not to come into contact with the unbearable emotions of the traumatized parts—the wall of illusion (see Fig. 2). In the case of involuntary childlessness, this is a particularly thick wall. It is reinforced by such lack of recognition from society—in the childless community this is often referred to as ‘disenfranchised grief’ (Day, 2021)—that the childless do not take it seriously themselves.

Although one could argue that a similar result could be achieved by attending a group of like-minded individuals, trauma therapy makes two distinctive contributions that are essential to reintegrating the survival- and traumatized parts. First, systematic therapy enables outlining the intention giver’s traumabiography. This is a process similar to drawing a map: the intention giver can clearly see how things connect over one’s own life experience, enabling both ownership of one’s experience, perspective, but also, just like a map, navigating forward. Second, it is through the resonance that one can go through the wall of the unconscious—making ‘those discoveries on another level than just thinking about things...reaching another level you were unaware of,’ as Liv put it—safely moving beyond the second wall: the portal to the trauma feelings that were once experienced as unbearable (see Fig. 2).

From a therapeutic perspective even 2 to 3 trauma-informed psychotherapy sessions did bring positive changes in the lives of the participants and help restore (at least some of) the moral agency they had lost. While I have not systematically compared IoPT with alternative therapeutic approaches, my argument is that if an experience is a trauma, it needs to be conceptualized and treated as such. This is why any treatment that involves mere affirmations or “rewriting” of one’s story—any approach that mostly revolves around the cognitive level (for one example: Aiyenigba et al., 2019)—is likely to have limited effects. It is crucial to address the roots of trauma by investigating one’s traumabiography. Where infertility turns into a self-inflicted trauma—for example by continuously subjecting oneself to invasive fertility treatments—or a re-traumatization, it is not sufficient to deal with infertility (an example of infertility-focused treatment is offered by Jaffe 2017), rather it is necessary to go back, often far back, into the individual’s past.

While this study confirms that the ability to tell and edit one’s story (Myers, 2016; Yahalom et al., 2023) is indeed crucial to repairing one’s moral agency—the outlining, in the therapy context, of the traumabiography is not merely a storytelling exercise. The charting of the traumabiography both supports and is the reflection of the integration of traumatized and survival parts that had split off. The integration of the parts is enabled not just by vocalizing the reality of what happened, but also by *feeling* it. Effectively, trying to change the story so that it is “empowering” and oriented toward “positive thinking” without an integration of the survival and traumatized parts, and a strengthening of the healthy parts consists in yet another dismissal of the reality of infertility and the emotions that revolve around it. Instead, understanding the experience of infertility, what it means, its existential repercussions, and especially how it is related to trauma is essential to providing a space where those who suffer can restore their moral agency: they feel listened to, safe and, ultimately, can begin the journey back to living fully.

## Conclusions

This study wanted to be a first step in taking the trauma of infertility seriously. I have shown, more specifically, that psychotrauma theory can help explain how infertility fits into one’s traumabiography. This provides answers to why for many individuals infertility is a trauma (i.e., a moral breakdown) in the first place; why the experience of infertility and what revolves around it, including fertility treatments, might be triggering of earlier traumas; why its grief is “complicated” and why it is so difficult, in the words of many of the individuals affected, to let go of the dream of having a child. The findings further indicate that the process of healing (i.e., moral agency repair) is supported by the outlining and vocalization of the traumabiography by the person affected by the erosion of moral agency; by her/his feeling, in the safe context of the therapy, of the emotions that were once unbearable; and by the non-judgmental validation and acknowledgment of the process by the therapist.

## Footnotes

1 Individuals who do not want children tend to self-identify as “childfree” (The Childfree Choice, 2022).

2 A review of the historical evolution of the concept of trauma is beyond the scope of this article. The reader might want to consult Ben-Ezra (2011) and Jongedijk et al. (2023). Bracken (2002) also contrasts positivist/cognitive understandings of “trauma” to approaches more oriented to meaning and phenomenology. This article embraces the latter.

3 Myers (2016, p. 430) refers to the ‘“breach” of life plan’ that leads to an erosion of moral agency with the Aristotelian term *peripeteia*.

4 People affected by PTSD may ‘relive the trauma’ through ‘flashbacks,’ ‘nightmares,’ avoiding ‘activities or places that recall the traumatic event,’ among other symptoms (APA, 2024).

5 Possible limitations relate to the small sample of participants, their being mostly white, heterosexual women all living in Norway, as well as the researcher sharing their same childless status. Based on previous research (Archetti, 2020), however, the experiences of the participants largely align with those of individuals (women and men) from other Western countries. There is also increasing demand for and acknowledgment of the value of research on involuntary childlessness produced by childless researchers (Archetti et al., 2023).
